# P-285. Hospitalization Patterns and ART Use Among Insured Adults with HIV in the United States, 2016–2021

**DOI:** 10.1093/ofid/ofaf695.506

**Published:** 2026-01-11

**Authors:** Daniel B Chastain, Xianyan Chen, Tonisha Gaitor, Hayley N Hemme

**Affiliations:** University of Georgia College of Pharmacy, Albany, GA; UGA Franklin College of Arts and Sciences, Athens, Georgia; University of Georgia College of Pharmacy, Albany, GA; University of Georgia, Athens, Georgia

## Abstract

**Background:**

Despite expanded access to antiretroviral therapy (ART), hospitalizations among people living with HIV (PWH) remain common, though the causes have shifted in the modern ART era. A better understanding of hospitalization patterns and the impact of ART is needed to inform targeted interventions. This study used insurance claims data to evaluate the incidence, causes, and predictors of hospitalization among insured PWH, with a focus on ART use.

**Table 1.**
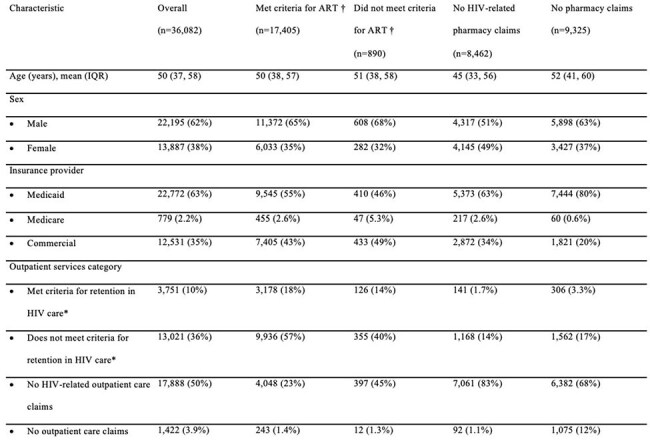
Baseline Characteristics at Index Hospitalization IQR, interquartile range *, Retention in HIV care was defined as ≥2 clinical encounters or laboratory results ≥3 months apart within the 6 months preceding the index hospitalization. †, ART use was determined through outpatient pharmacy claims using National Drug Codes (NDCs) prior to hospitalization.

**Table 2.**
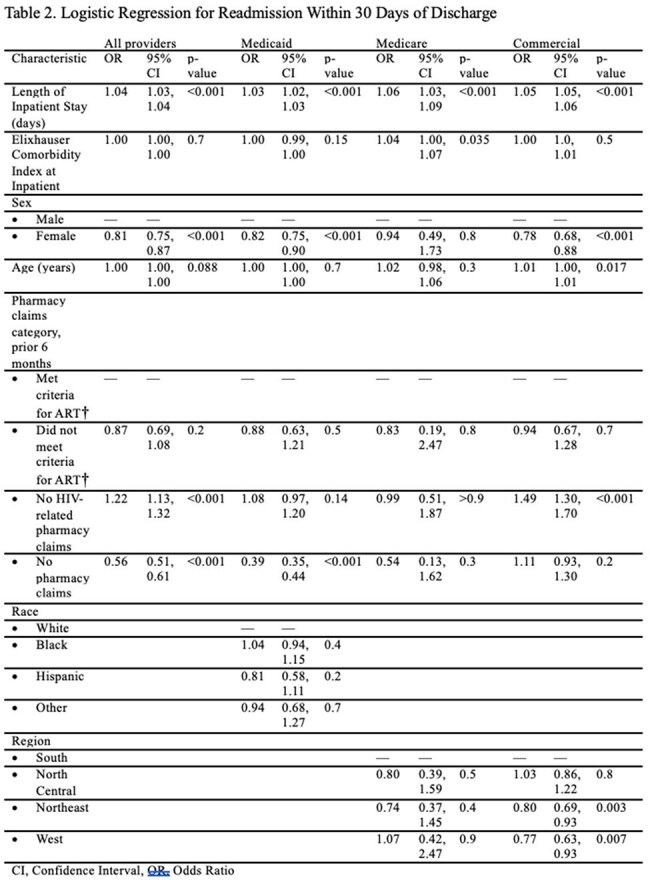
Logistic Regression for Readmission Within 30 Days of Discharge CI, Confidence Interval, OR, Odds Ratio †, ART use was determined through outpatient pharmacy claims using National Drug Codes (NDCs) prior to hospitalization.

**Methods:**

This retrospective cohort study analyzed data from adults with HIV (ICD-10-CM code B20) using MarketScan (2016-2021). Inclusion required continuous insurance enrollment for ≥6 months before the index hospitalization and a minimum of 30 days post-discharge for survivors. Causes of hospitalization and engagement in outpatient HIV care were compared between those on ART and those not on ART. Predictors of 30-day readmission were evaluated using multivariable logistic regression, adjusting for demographic and clinical factors.

**Figure 1.**
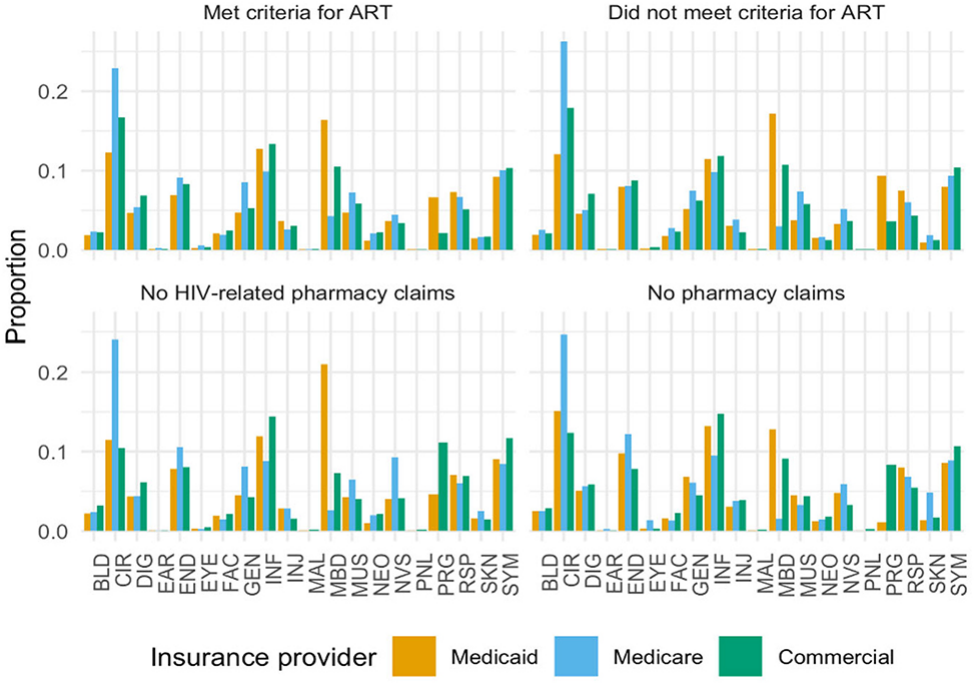
Clinical Classifications Software Refined (CCSR) Categories Stratified by Pharmacy Claims Categories and Insurance Providers

Proportion of Clinical Classifications Software Refined (CCSR) categories by pharmacy claim category and insurance provider. The y-axis shows the proportion, the x-axis displays CCSR categories, and facets represent pharmacy claim categories. Bar colors indicate insurance providers, illustrating the distribution of CCSR categories within each pharmacy claim and insurance provider combination. BLD, Diseases of the blood and blood-forming organs and certain disorders involving the immune mechanism; CIR, Diseases of the circulatory system; DEN, Dental diseases; DIG, Diseases of the digestive system; EAR, Diseases of the ear and mastoid process; END, Endocrine, nutritional and metabolic diseases; EXT, External causes of morbidity; EYE, Diseases of the eye and adnexa; FAC, Factors influencing health status and contact with health services; GEN, Diseases of the genitourinary system; INF, Certain infectious and parasitic diseases; INJ, Injury, poisoning and certain other consequences of external causes; MAL, Congenital malformations, deformations and chromosomal abnormalities; MBD, Mental, behavioral and neurodevelopmental disorders; MUS, Diseases of the musculoskeletal system and connective tissue; NEO, Neoplasms; NVS, Diseases of the nervous system; PNL, Certain conditions originating in the perinatal period; PRG, Pregnancy, childbirth and the puerperium; RSP, Diseases of the respiratory system; SKN, Diseases of the skin and subcutaneous tissue; SYM, Symptoms, signs and abnormal clinical and laboratory findings, not elsewhere classified.

**Figure 2.**
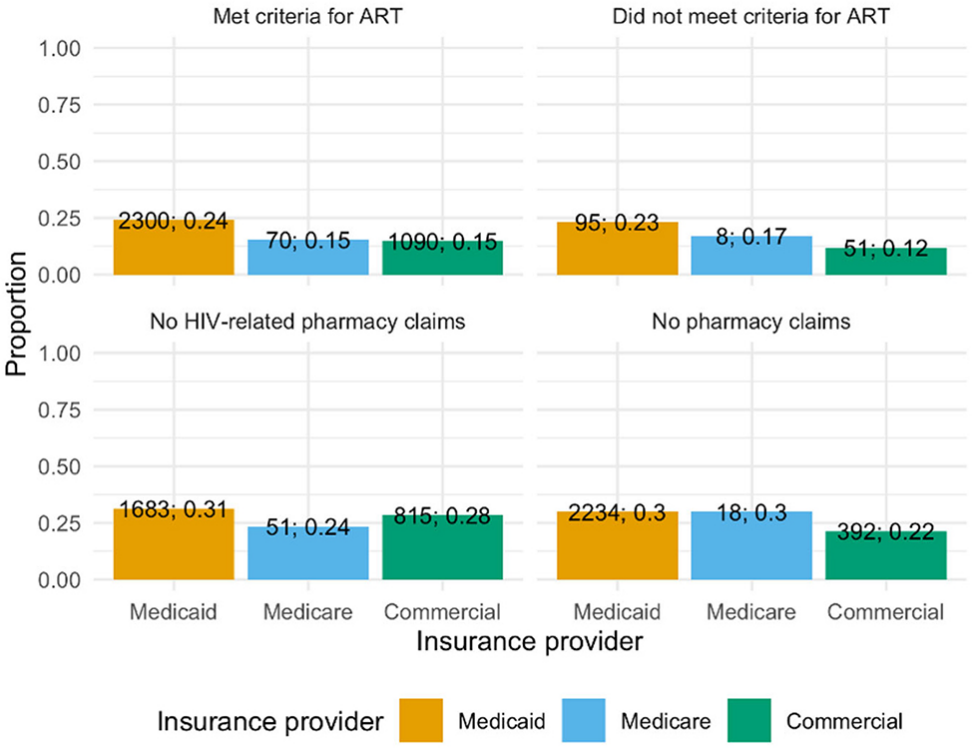
Proportion with Any HIV-Associated Syndrome at Index Hospitalization by Pharmacy Claims Category and Insurance Provider

Proportion of individuals with 30-day readmission, stratified by insurance provider and pharmacy claim category. The y-axis displays the proportion of readmissions. Insurance provider is indicated by the x-axis and bar color. Facets represent pharmacy claim categories. Numbers above bars show the total number of enrollees and the proportion with 30-day readmission for each group.

**Results:**

A total of 36,082 PWH were included. Most patients were male (62%) and had a substantial comorbidity burden (Table 1), with the majority insured through Medicaid (63%). Among the cohort, 48% were prescribed ART prior to admission. The most common causes of hospitalization were circulatory diseases (14%), mental, behavioral, and neurodevelopmental disorders (14%), and infectious diseases (13%), with similar distributions across ART claim categories (Fig 1). Only 24% of hospitalizations were attributed to HIV-associated conditions, most frequently HCV and tuberculosis. The proportion of HIV-associated conditions was highest among those without HIV-related pharmacy claims or no pharmacy claims and lowest among those prescribed ART (Fig 2). In the overall cohort, longer inpatient stays were significantly associated with higher odds of 30-day readmission (OR 1.04 per day, p< 0.001) (Table 2). Patients with no HIV-related pharmacy claims had greater odds of readmission compared to those prescribed ART (OR 1.22, p< 0.001).

**Conclusion:**

Hospitalizations among insured PWH were largely due to non–HIV-related conditions, with gaps in ART use and outpatient care contributing to worse outcomes. Lack of ART was associated with higher rates of HIV-related admissions and increased 30-day readmission risk.

**Disclosures:**

All Authors: No reported disclosures

